# Heart disease in women: the role of imaging

**DOI:** 10.1007/s12471-019-1265-y

**Published:** 2019-04-04

**Authors:** P. van der Bijl, V. Delgado, J. J. Bax

**Affiliations:** Department of Cardiology, Heart Lung Centre, Leiden University Medical Centre, Leiden, The Netherlands

Cardiovascular disease is the leading cause of death in European women. In addition, a greater percentage of women die from cardiovascular disease than do men (49% vs 45%, respectively). Certain cardiac diseases occur more often in women, and the essential diagnostic role of cardiac imaging will be illustrated by way of three representative conditions: (1) coronary microvascular disease (CMVD), (2) takotsubo cardiomyopathy and (3) mitral valve prolapse (MVP).

**Fig. 1a–d:**
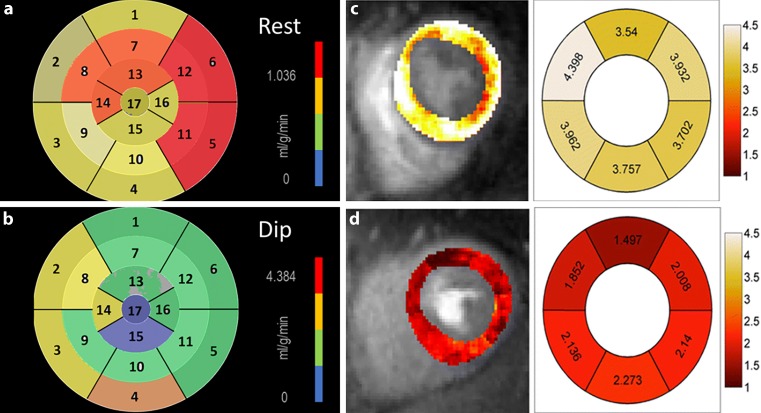
Coronary microvascular disease imaging. Positron emission tomography (^13^N-ammonia) myocardial perfusion in a patient with unobstructed coronary arteries on invasive angiography, showing normal (**a**) blood flow before and decreased (**b**) blood flow after dipyridamole (*Dip*). Cardiac magnetic resonance myocardial perfusion maps demonstrating reduced blood flow after regadenoson (**d**), compared to a normal individual (**c**). Absolute myocardial blood flow (ml/g per minute) is reduced in all six segments of the short-axis slice after pharmacological stress. **c**, **d** Reproduced from Zorach et al. [2] with permission

More than half of women with chronic chest pain who undergo anatomic coronary artery imaging (e. g. invasive coronary angiography) do not show obstructive, epicardial coronary artery disease [1]. Many of them demonstrate inducible ischaemia on functional testing (e. g. stress electrocardiography (ECG)), and also experience an impaired quality of life and an increased risk of adverse cardiovascular events [1]. This discrepancy between functional and anatomical imaging was often attributed to false-positive stress testing, but it is increasingly recognised that CMVD with abnormal endothelial and microvascular function is the underlying cause [1]. Non-obstructive epicardial coronary artery disease could also account for some women with chest pain symptomatology, and this can be diagnosed non-invasively with computed tomography angiography (CTA) [1]. The spatial resolution of CTA is inadequate for the diagnosis of CMVD, and the gold standard is represented by invasive measurement of coronary microvascular resistance. This, however, is a time-consuming and complex technique. Various cardiac imaging modalities are promising tools that can diagnose CMVD non-invasively. Impaired coronary flow reserve or decreased myocardial perfusion can be demonstrated in patients with CMVD using transthoracic echocardiography, positron emission tomography (Fig. 1a and b), dynamic myocardial perfusion computed tomography and cardiac magnetic resonance (CMR) imaging ([2]; Fig. 1c and d). Cutting-edge techniques, e. g. CMR stress T1 mapping, have been validated against invasively measured fractional flow reserve and index of microvascular resistance (as gold standards of epicardial coronary disease and CMVD, respectively) [3].

Another cardiovascular disease which is more frequent in women than in men is takotsubo cardiomyopathy, an acute heart failure syndrome, usually following a severe physical or psychological stressor [4]. Takotsubo cardiomyopathy is diagnosed in about 8% of women who present with a suspected acute coronary syndrome, compared to less than 1% in men [5]. The clinical profile is similar to an acute coronary syndrome: chest pain, ischaemic ECG changes and regional left ventricular wall motion abnormalities [4]. While echocardiography and left ventriculography demonstrate a regional wall motion abnormality (typically mid-to-apical systolic dyskinesia, extending beyond a single coronary artery territory), unobstructed vessels on invasive coronary angiography do not reliably distinguish a spontaneously reperfused acute coronary syndrome from takotsubo cardiomyopathy. Myocardial inflammation (oedema on T2-weighted CMR images) can be visualised in both takotsubo cardiomyopathy and acute coronary syndromes, but the presence of delayed myocardial enhancement after contrast administration usually indicates the presence of myocardial infarction and allows differentiation of these two entities [4].

Furthermore, MVP is more common in women and is characterised by a number of gender differences in mitral valve morphology, i. e. prolapse involving more often the anterior segments or both leaflets, more thickened leaflets, and fewer instances of flail leaflets [6]. Moreover, women develop smaller absolute left atrial and left ventricular size increases as a consequence of mitral regurgitation, although their left atrial and left ventricular sizes are larger than in their male counterparts, when normalised to body surface area [6].

Multimodality cardiac imaging modalities have a pivotal role in diagnosing women with suspected heart disease. Imaging techniques and parameters for the diagnosis of CMVD require further validation against invasive measurements, as well as comparison of the diagnostic accuracy of the different non-invasive techniques. The current treatment of CMVD is purely empirical, and reliable non-invasive imaging techniques are expected to underpin therapeutic outcome trials in the future. Imaging is also likely to provide better insight into the risk stratification of cardiac diseases which occur predominantly in women. In the contemporary era of personalised (and precision) medicine, practising gender-specific cardiology is highly relevant, and this principle should be integrated into the selection and interpretation of cardiac imaging investigations.
